# Bacterial Adhesion to Natural and Synthetic Fibre-Forming Polymers: Influence of Material Properties

**DOI:** 10.3390/polym16172409

**Published:** 2024-08-24

**Authors:** Nina Čuk, Barbara Simončič, Rok Fink, Brigita Tomšič

**Affiliations:** 1 Faculty of Natural Sciences and Engineering, University of Ljubljana, Aškerčeva cesta 12, 1000 Ljubljana, Slovenia; nina.cuk@ntf.uni-lj.si; 2 Faculty of Health Sciences, University of Ljubljana, Zdravstvena pot 5, 1000 Ljubljana, Slovenia; rok.fink@zf.uni-lj.si

**Keywords:** bacterial adhesion, textile factors, anti-adhesive polymer-based textiles, anti-adhesion surface

## Abstract

Polymer-based textiles have a major impact on human well-being, as they provide the desired functional protection and aesthetic comfort when worn. However, natural and synthetic polymer-based textiles can also pose serious health risks, as they are surfaces that allow the adhesion of various bacteria, including pathogenic bacteria. To minimise these problems, antibacterial chemical treatments are generally applicable in the case of polymer-based textiles. However, to avoid the use of potentially toxic chemicals, sustainable approaches require the customised design of non-adhesive polymer-based textiles, considering their chemical, physicochemical, constructional, and textural properties. Before designing, several articles are required to gain sufficient knowledge of the described object. Despite the urgent need to combat bacteria (on polymer-based textiles), which pose a serious global health risk, only a few review articles have been published that address bacterial adhesion in the context of superhydrophobic and antibacterial textile materials, while only one review article holistically addresses the textile factors and their influence on this phenomenon. The aim of this review article is to expand the insufficient knowledge about bacterial adhesion to polymer-based textiles on the basis of theoretical findings and real examples through a high degree of structuring, simplification, holistic consideration, and visualization. Therefore, this review provides an insight into the mechanisms involved in bacterial adhesion and a comprehensive overview of the influence of different textile factors, such as chemical composition, hydrophilicity/hydrophobicity, surface charge, surface free energy, roughness, and porosity, on bacterial adhesion. To emphasise the importance of the synergistic effect of the combined textile factors, examples of the influence of hydrophilicity/hydrophobicity in combination with surface charge, surface roughness, and porosity are discussed. From the review, it can be concluded that the combination of hydrophilicity/hydrophobicity and the surface charge of textile fibres and bacteria is crucial for bacterial adhesion, with roughness and porosity being the most important factors among the constructive and textural properties of polymer-based textiles.

## 1. Introduction

Humans are in constant dynamic contact with textile material via their skin [1,2,3]. Due to their unique chemical, morphological, and structural properties, polymer-based textiles are a suitable solid surface for bacterial adhesion and biofilm growth [4,5,6,7]. Bacteria can survive on various types of polymer-based textiles for up to 206 days, meaning that they remain a source of transmission for weeks [8]. This can impair the functionality of the material and, in the worst case, lead to serious health problems for users. In terms of negative effects on textile materials, the presence of bacteria on polymer-based textiles can deteriorate their aesthetic, functional, and mechanical properties due to staining, discolouration, and fibre degradation [9]. The presence of bacteria on polymer-based textiles, which can be transferred from fabric to skin [10] or from fabric to fabric [11], is not only a pressing problem in hospital environments, where it poses a high risk of causing various infections [12], but also in everyday life, as potentially pathogenic microorganisms on polymer-based textiles can also be transferred to household environments [13]. Bacterial infections have been found to be a clinically significant cause of health loss worldwide. Five pathogens (*Staphylococcus aureus*, *Escherichia coli*, *Streptococcus pneumoniae*, *Klebsiella pneumoniae*, and *Pseudomonas aeruginosa*) were involved in more than 500,000 deaths in 2019 [14]. Bacterial resistance to antibiotics has recently proven to be particularly problematic and, according to the World Health Organization (WHO), poses a growing threat to public health that is relevant to many countries and various sectors [15]. Nevertheless, bacterial resistance to antibiotics in biofilms (a form that provides protection from hostile environmental conditions) is even more problematic, as it plays an important role in the pathogenesis of numerous subacute and chronic bacterial diseases, especially infections associated with medical devices [16].

**Figure 1:**
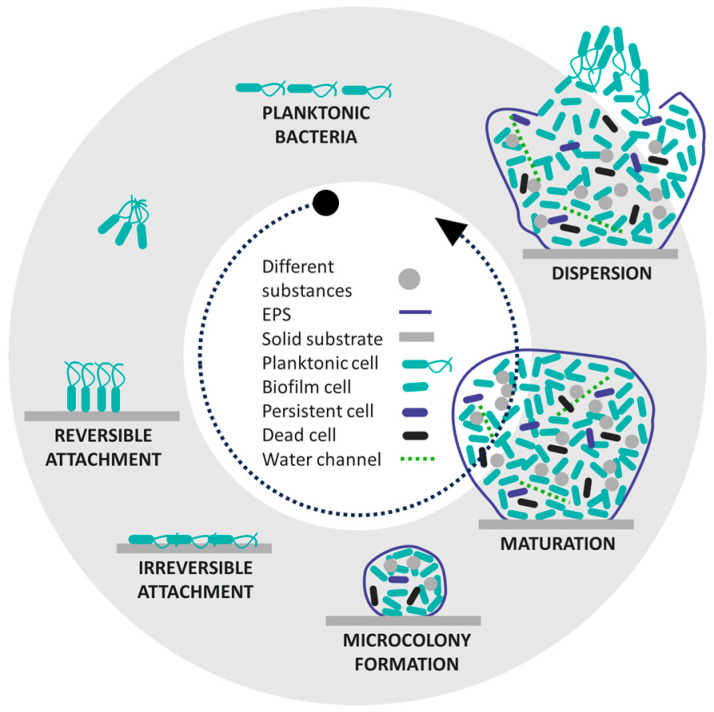
Five-step process of developing the biofilm. Reproduced with permission from [38], copyright 2021, Springer Nature.

It is known that bacterial cells tend to grow on solid surfaces and not in the surrounding aqueous phase [17]. They can multiply very rapidly under suitable conditions, determined by humidity, temperature, and the presence of nutrients [4,5,7,18,19]. Biofilm development occurs across several steps, which have been thoroughly studied for decades [4,5,7,18,19,20,21,22,23,24,25,26,27,28,29,30,31,32,33,34,35,36,37,38,39,40,41,42]. The process begins with the formation of a conditioned film on the surface of a solid substrate, followed by the migration of bacteria to the immediate vicinity of the surface and their reversible and irreversible adhesion to the conditioned surface. Reversible adhesion is the initial weak attachment of bacterial cells to a surface, while irreversible adhesion involves the permanent binding of bacteria to the surface with the help of specific bacterial adhesins. The adhering bacteria then begin to grow and divide, leading to the formation of microcolonies, extracellular polymeric substances (EPSs), and later, biofilm. The final phase is the disintegration of the biofilm when the EPSs open and the cells are released and colonise new surfaces (Figure 1) [30,31,36,43,44,45].

**Figure 2:**
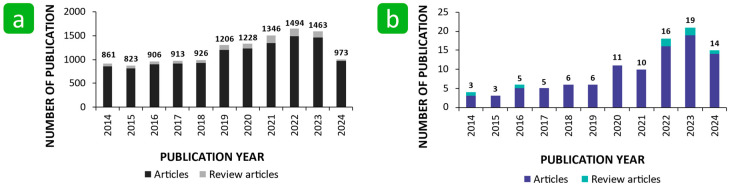
The number of publications related to bacterial adhesion in general (**a**) and in relation to textiles (**b**) since 2014, using the words in the abstract and topic: (**a**) for articles AB = (“bacterial adhesion” OR “bacterial adherence”), and review articles AB = (“bacterial adhesion” OR “bacterial adherence”) AND TS = review; (**b**) for articles AB = (“bacterial adhesion” OR “bacterial adherence”) AND AB = (textile* OR fabric), and review articles (Source Web of Science, date of search: 30 July 2024).

To avoid the problems caused by the growth of bacteria on solid surfaces, including natural and synthetic polymer-based textiles, understanding bacterial adhesion is crucial, as this step represents the beginning of colonisation and biofilm formation. The importance of this research topic across various fields is evidenced by the literature, in which over 20,000 articles on bacterial adhesion have been published, including over 1400 review articles (Figure 2a) (source: Web of Science). Despite the great interest in this research topic, the number of articles dealing with bacterial adhesion to textile surfaces is significantly lower, which means that this area of research is still largely unexplored (Figure 2b). However, the number of publications has gradually increased over the last ten years, with a significant increase in recent years due to concerns about the COVID-19 pandemic.

Furthermore, to our knowledge, only seven review articles have been published to date that deal with bacterial adhesion to textile substrates (Web of Science). A look at these review articles shows that most of them provide a systematic overview of recent research on superhydrophobic and antibacterial textile materials. They focus on different strategies to produce superhydrophobic rough surfaces with anti-adhesion and antibacterial properties and their potential applications [46,47], as well as on the development of textile fabrics for medical applications that are able to absorb liquids but do not adhere to wounds or favour the growth of biofilms [48], on textile surfaces with active antimicrobial coatings produced using various conventional organic and novel inorganic antibacterial agents at the nanoscale and their antibacterial efficacy [49], and on textile materials for the application of antibacterial hydrogel dressings for the treatment of infected and chronic wounds [50]. Only one review article focused on bacterial adhesion to textile surfaces in relation to factors that influence this phenomenon, such as the surface properties of the bacteria and textile fabrics, as well as environmental factors [51]. To develop guidelines for the adaptation of textile materials that minimise the risk of nosocomial infections from polymer-based textiles, an in-depth understanding of the various factors that influence the interactions between polymer-based textiles and microbes is of great importance. The coverage of the different aspects of the same main topic was therefore ensured in this review article through interdisciplinary work, interconnecting textile engineering, physiochemistry, and microbiology.

To highlight the importance of textile factors, this review article aims to provide a comprehensive overview of the influence of the chemical and physicochemical properties of textile fibres and the constructional and textural properties of fabrics on bacterial adhesion, with a focus on the chemical composition of fibres, surface charge, hydrophilicity/hydrophobicity, surface free energy, surface roughness, and porosity. The theoretical mechanisms of bacterial adhesion are also summarised. The complexity of bacterial adhesion as a function of textile factors is illustrated using examples in which the combined chemical, physicochemical, constructional, and textural factors are analysed in relation to bacterial adhesion. These results are discussed in comparison to other solid substrates. Prospects and research directions for future investigations focusing on the interactions between bacteria and textile surfaces are also addressed. To summarize, this article presents a sensitive topic holistically by considering various aspects/theories and interweaving these theoretical foundations with concrete investigations and examples, firstly in the world of textiles and secondly in a broader sense with other materials. Despite the complexity of the topic and the multi-layered presentation, the article retains its structure and simplicity. We believe that this review will serve as both a theoretical (treasure trove) and practical (source of inspiration) basis for further research and development in the field of bacterial control, which cannot be easily transferred from other areas due to the unique properties and behaviours of polymer-based textiles. By considering various textile factors in the context of bacterial adhesion, the focus is already taken into account at the beginning of the textile production process and not only at the end of production (i.e., during various finishing processes), as is common today. Namely, optimising textile factors and disrupting the balance of attractive and repulsive forces could result in reduced bacterial attachment, which would be useful in creating antimicrobial fabrics for healthcare, sportswear, and food processing applications.

## 2. Theoretical Approach of Bacterial Adhesion

**Figure 3:**
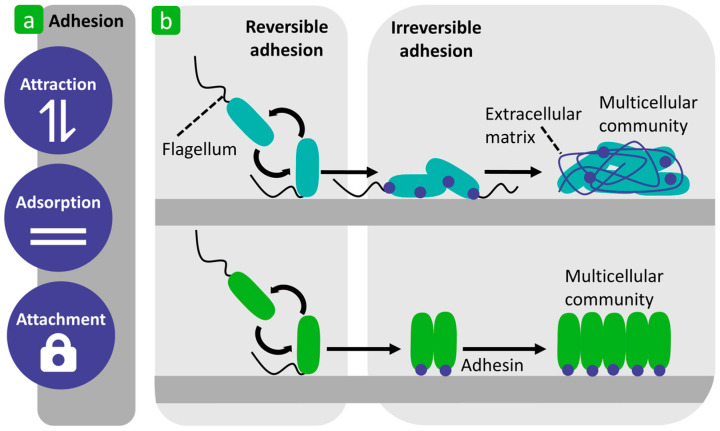
Schematic representation of bacterial adhesion. (**a**) Definition. (**b**) Two different forms of multicellular communities through extracellular matrix secretion and the secretion of adhesion. Reproduced with permission from [61], copyright 2019, Springer Nature.

Bacterial adhesion describes a very complex process involving the attraction, adsorption, and attachment of bacterial cells to solid substrates from a liquid environment [52,53]. This process can be divided into two phases: reversible adhesion (referred to as the docking phase) and irreversible adhesion (referred to as the locking phase) (Figure 3) [22,54,55,56,57,58,59,60,61,62,63,64].

Reversible adhesion represents the initial phase of the process of bacterial adhesion and involves the immediate attraction of planktonic bacteria to a substrate surface, which occurs when the bacteria enrich the surface of the substrate by passive and/or active movement, but still exhibit Brownian motion (the phenomenon refers to the random movement of a particle in a medium [65]) [54,58,60,61,66]. In this phase, different attractive (van der Walls) and repulsive (electrostatic) physicochemical forces occur between the surfaces of the bacteria and the substrate, which depend on the chemical structures of the surfaces and the properties of the surrounding liquid. When the attractive forces outweigh the repulsive forces, the process of bacterial adhesion progresses [60,61].

In irreversible adhesion, the bacteria, which no longer show Brownian movement, adhere firmly to the substrate surface and strengthen their adhesion through molecular and cellular interactions and through the production of specific adhesin molecules (the role of specific proteinaceous adhesions in bacteria is to attach to surfaces and live in close association with them [67]) [58,60]. In this phase, the bacteria adhere permanently to the surface by producing exopolysaccharides (they have a wide range of chemical structures and two forms, capsular or slime, whose function is to protect bacteria [68]) that form a complex with the substrate surface and/or receptor-specific ligands located on pili, fimbriae, and fibrillae, or both. The bacterial cells can also adhere to each other and form aggregates on the substrate [60]. The characteristics of the two mechanisms of bacterial adhesion, namely reversible and irreversible, are shown in Table 1.

**Figure 4:**
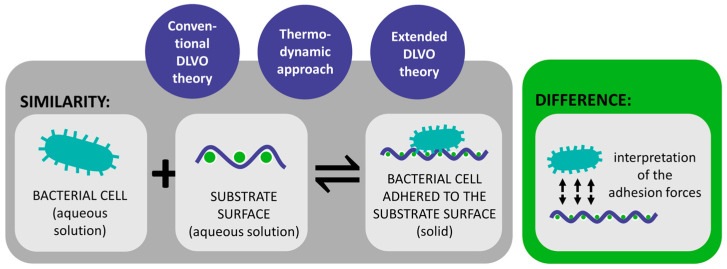
Similarities and differences between the conventional DLVO theory, thermodynamic approach, and extended DLVO theory.

Three theoretical approaches have been used to describe the physicochemical interactions involved in the process of bacterial adhesion: the conventional Derjaguin–Landau–Verwey–Overbeek (DLVO) theory [53], the thermodynamic approach [69], and an extended DLVO theory [70]. All three theoretical approaches describe bacterial adhesion as a reversible process in thermodynamic equilibrium [58] but differ in the interpretation of the associated adhesion forces (Figure 4) [18].

The conventional DLVO theory, originally developed to explain the long-range forces between colloidal particles in a solution, and which is of great importance in colloid and surface chemistry [71], has also been applied to describe bacterial adhesion to substrate surfaces, since bacteria are almost as large as colloidal particles and can be considered inert colloidal particles [53,54]. Bacterial adhesion is assumed to be balanced by two main forces between the bacterial cells and the substrate surface, i.e., attractive Lifshitz–van der Waals forces and repulsive or attractive electrostatic forces, which are distance-dependent and decrease with increasing distance [18,29,72,73,74,75]. Accordingly, the energy of bacterial adhesion can be expressed as follows [18]:

(1) $$G_{BLS}^{DLVO}(d)=G_{BLS}^{LW}(d)+G_{BLS}^{EL}(d)$$

where $G_{BLS}^{DLVO}$ represents the total interaction energy between the bacterium and the substrate surface immersed in liquid, *d* denotes the distance dependence, $G_{BLS}^{LW}$ describes the Lifshitz–van der Waals interactions, and $G_{BLS}^{EL}$ represents the electrostatic interactions. The latter results from the electrical double layer that occurs at the interface between the charged surface of the particles and a surrounding liquid and influences the strength of the electrostatic interactions [71]. Since most colloidal particles [76,77] and solid surfaces, including textile substrates [78,79,80], carry the same net negative charge in water at a neutral pH level, the electrostatic forces are normally repulsive [29,53,54,81,82,83,84].

**Figure 5:**
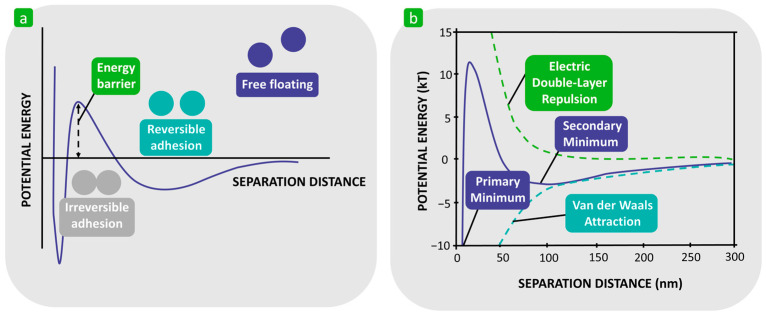
Schematic presentation of conventional DLVO theory of bacterial adhesion in different stages. (**a**) Reversible and irreversible adhesion in terms of potential energy and separation distance. Reproduced with permission from [85], copyright 2013, the Royal Society of Chemistry. (**b**) Representation of secondary and primary minimum as well as electric double-repulsion and van der Waals attraction. Reproduced with permission from [58], copyright 2000, Springer Nature.

Figure 5 shows the potential energy of the interaction between the bacterial cell, described as a charged colloidal particle, and the same charged flat substrate surface as a function of distance [58,85,86]. This shows that the sum of the Lifshitz–van der Waals and the electrostatic double layer interactions has two minima of interaction potential energy, a secondary and a primary minimum. The secondary minimum, which results from the balance between long-range attractive and repulsive forces away from the contact, is at distances of up to 100 nm, and as long as the bacteria are within this interaction minimum, reversible bacterial adhesion exists. To reach the primary potential energy minimum, which results from a balance between short-range attractive and repulsive forces at the point of molecular contact and where irreversible bacterial adhesion is possible, the bacteria should have the activation energy to overcome the potential energy barrier that lies between the two minima [58,72,87,88,89]. The height of the potential barrier is influenced by the Brownian thermal flocculation rate and the surface potential [71] and, theoretically, the rate at which the bacteria overcome the energy barrier decreases exponentially with the height of the energy barrier [58].

The DLVO theory of bacterial adhesion has some limitations, as it does not take into account that bacteria are much more complex than inert spherical colloid particles and that bacterial adhesion is directly influenced by various factors, such as the ionic strength and the pH of the solution, the presence of specific adhesions on the bacterial surface, the adsorption of components of the conditioning film on the substrates, and the roughness of the substrates [29,90,91].

**Figure 6:**
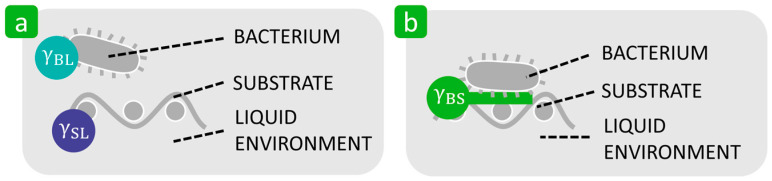
Schematic representation of the thermodynamic approach based on the interfacial free energy balance. (**a**) Existence of the bacteria–liquid and substrate–liquid interfaces before bacterial adhesion. (**b**) Existence of the bacteria–substrate interface after bacterial adhesion.

The thermodynamic approach explains bacterial adhesion to solid substrates based on the interfacial free energy balance [18,29,69,92]. In the adhesion process, a new interface between bacteria and a substrate is created by interrupting two existing interfaces, namely the interfaces between the bacteria and liquid and between the substrate and liquid (Figure 6).

The tendency of bacterial adhesion is expressed by the interfacial Gibs free energy of adhesion, ${\Delta G}_{BLS}^{adh}$, per unit area of a bacterium on the substrate surface in a suspension liquid, according to the following equation:

(2) $${\Delta G}_{BLS}^{adh}=\gamma_{BS}-\gamma_{BL}-\gamma_{SL}$$

where $\gamma_{BS}$ stands for the bacteria–substrate interfacial free energy, $\gamma_{BL}$ represents the bacteria–liquid interfacial free energy, and $\gamma_{SL}$ denotes the substrate–liquid interfacial free energy. Since, according to the second law of thermodynamics, the spontaneous process is accompanied by a decrease in the Gibs free energy of the system, adhesion is favourable if ${\Delta G}_{BLS}^{adh}<0$ and unfavourable when ${\Delta G}_{BLS}^{adh}>0$ [18,29]. If the work of adhesion, *Wa*, is used instead of the Gibs free energy as a measure of the strength of bacterial adhesion, it should be noted that bacterial adhesion increases with increasing Wa, because $-{\Delta G}_{BLS}^{adh}=Wa$ [93].

Different thermodynamic approaches can be used to calculate ${\Delta G}_{BLS}^{adh}$ [94]. These generally include the dispersion–polar approach proposed by Fokes, Owens, and Wendt [95,96,97,98] and the Lifshitz–van der Waals acid–base approach proposed by van Oss [70,99,100].

The dispersion–polar approach expresses ${∆G}^{adh}$ as the sum of the dispersion component, ${∆G}^{d}$, and the polar component, ${∆G}^{p}$, as follows [96]:

(3) $${∆G}^{adh}={∆G}^{d}+{∆G}^{p}$$

where, as an approximation, the dispersion interactions mainly comprise the attractive London dispersion forces and the polar interactions mainly comprise the hydrogen bonds.

The Lifshitz–van der Waals acid–base approach expresses ${∆G}^{adh}$ as the sum of the Lifshitz–van der Waals, ${∆G}^{LW}$, and acid–base, ${∆G}^{AB}$, adhesion energies according to the following equation [101]:

(4) $${∆G}^{adh}={∆G}^{LW}+{∆G}^{AB}$$

where the Lifshitz–van der Waals interactions are non-polar and comprise predominantly London dispersion forces and the acid–base interactions are polar and comprise two non-additive parameters, i.e., the electron acceptor parameter labelled $\gamma^{+}$ (Lewis acid) and the electron donor parameter labelled $\gamma^{-}$ (Lewis acid) [18,102,103].

According to the literature, both the conventional DLVO theory and the thermodynamic approach have not been entirely successful in predicting and explaining the different adhesion behaviours due to the complexity of the bacterial adhesion process [53,72,75]. To overcome the limitations of the DLVO theory and describe the polar interactions in water, the extended DLVO theory was proposed [70,102,104]; the energy component, $U_{BLS}^{AB}$, which describes the acid–base interactions, is included in Equation (5) in addition to the Lifshitz–van der Waals and electrostatic double-layer interactions. Accordingly, the total adhesion energy in the extended DLVO theory can be expressed as follows:

(5) $$G_{BLS}^{XDLVO}(d)=U_{BLS}^{LW}(d)+U_{BLS}^{EL}(d)+U_{BLS}^{AB}(d)$$

where $U_{BLS}^{AB}$ describes attractive hydrophobic interactions or repulsive hydrophilic or hydration effects that depend on the hydrophobic/hydrophilic properties of both the bacteria and the substrate surfaces [29,75,105]. It has been shown that the extended DLVO approach generally predicts adhesion and its reversibility more accurately than the DLVO theory and the thermodynamic approach [29,75,89,105].

To apply the thermodynamic and the extended DLVO approaches for the quantitative investigation and prediction of bacterial adhesion, the thermodynamic surface properties of bacteria, substrate, and liquid medium should be determined independently of each other [73,105,106]. While the thermodynamic surface properties of liquids (*L*) can be measured directly in terms of surface tension, $\gamma_{L}$, the thermodynamic surface properties of bacteria and substrate can be determined indirectly by measuring the contact angle of different polar and non-polar liquids on their surfaces and using the Lifshitz–van der Waals acid–base approach [107]. This approach assumes that the interfacial free energy between two moieties, i.e., *i* and *j*, is expressed by the surface free energies of the moieties:

(6) $$\gamma_{ij}=\gamma_{i}+\gamma_{j}-2\sqrt{\gamma_{i}\gamma_{j}}$$

and that the surface free energy of the moiety, for example *i*, is expressed by its non-polar and polar components as follows:

(7) $$\gamma_{i}=\gamma_{i}^{LW}+\gamma_{i}^{AB}=\gamma_{i}^{LW}+2\sqrt{\gamma_{i}^{+}\gamma_{i}^{-}}$$

where $\gamma^{LW}$ represents the Lifshitz–van der Waals component of the surface free energy, $\gamma^{+}$ denotes the electron–acceptor parameter, and $\gamma^{-}$ indicates the electron donor parameter of the Lewis acid–base component, $\gamma^{AB}$, of the surface free energy.

Taking all of these assumptions (Equations (6) and (7)) into account, the ${\Delta G}_{BLS}^{adh}$ of a bacterium (*B*) on a substrate (*S*) surface in a suspension liquid (*L*) in Equation (2) is expressed as follows [92,102]:

(8) $$\begin{array}{c} \Delta G_{BLS}^{adh}=2[\sqrt{\gamma_{B}^{LW}\gamma_{L}^{LW}}+\sqrt{\gamma_{S}^{LW}\gamma_{L}^{LW}}-\sqrt{\gamma_{B}^{LW}\gamma_{S}^{LW}}-\gamma_{L}^{LW}+\sqrt{\gamma_{L}^{+}}\left(\sqrt{\gamma_{B}^{-}}+\sqrt{\gamma_{S}^{-}}-\sqrt{\gamma_{L}^{-}}\right)+\\ \sqrt{\gamma_{L}^{-}}\left(\sqrt{\gamma_{B}^{+}}+\sqrt{\gamma_{S}^{+}}-\sqrt{\gamma_{L}^{+}}\right)-\sqrt{\gamma_{B}^{+}\gamma_{S}^{-}}-\sqrt{\gamma_{B}^{-}\gamma_{S}^{+}}] \end{array}$$

The values of the surface free energy of the solid (bacterium or substrate) and its components in Equation (8) can be determined experimentally by introducing the Young–Dupre’ equation. The latter expresses the relationship between the liquid–solid contact angle, *θ*, measured with drops of the liquid of $\gamma_{L}$ deposited on the solid of $\gamma_{S}$ using the following equation [107]:

(9) $$\gamma_{L}\left(1+\cos\theta \right)=-{\Delta G}_{SL}=2\left(\sqrt{\gamma_{S}^{LW}\gamma_{L}^{LW}}+\sqrt{\gamma_{S}^{+}\gamma_{L}^{-}}+\sqrt{\gamma_{S}^{-}\gamma_{L}^{+}}\right)$$

In the experiment, the liquid contact angle on the surfaces of the bacteria and the substrate is measured goniometrically in thermodynamic equilibrium with three different liquids, including one non-polar and two polar liquids.

To conclude the theoretical part of this review article, the strengths, limitations, and applications of the three theories presented are summarised in Table 2.

## 3. Textile Factors Influencing Bacterial Adhesion

**Figure 7:**
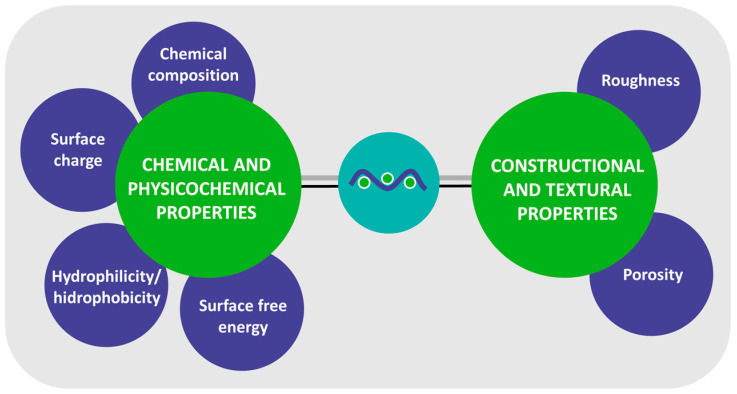
The main textile factors influencing bacterial adhesion.

Textile factors that influence bacterial adhesion can be divided into two groups: (i) chemical and physicochemical properties and (ii) constructional and textural properties (Figure 7). Chemical and physicochemical properties include the chemical structure of the fibres, surface charge, hydrophilicity/hydrophobicity, and surface free energy. The most important constructional and texture properties that influence bacterial adhesion are surface roughness and porosity. These factors are discussed in detail in this section.

### 3.1. Chemical and Physicochemical Properties

**Figure 8:**
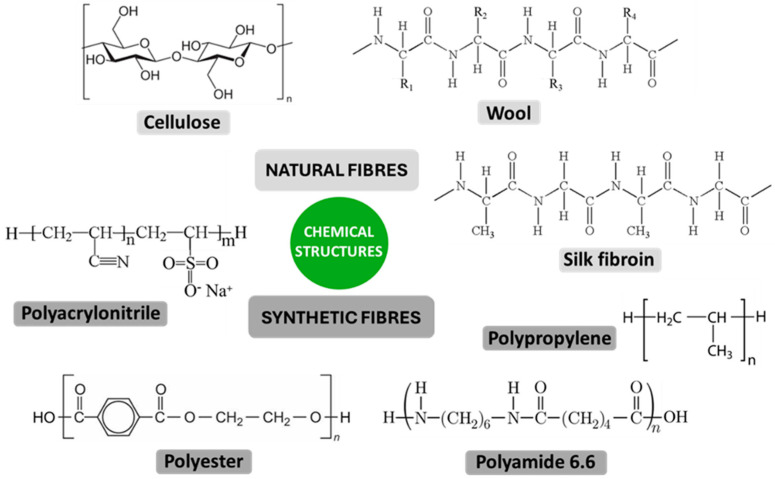
Chemical structures of common natural and synthetic fibre-forming polymers.

When discussing the influence of the chemical structure, hydrophilicity/hydrophobicity, surface charge, and the surface free energy of textile fibres on bacterial adhesion, it should be emphasised that these factors are highly interdependent, as the chemical structure and the presence of functional groups in the natural and synthetic fibre-forming polymers determine their hydrophilicity and surface charge. In addition, hydrophilicity is directly related to the surface free energy of the fibres, as fibres that are more hydrophilic have higher surface free energy. Textile fibres are formed of natural or chemical fibre-forming polymers with different chemical structures. The most important natural fibres include cotton, linen, hemp, jute, wool, and silk, while chemical synthetic fibres include polyester, polyamide, polypropylene, and polyacrylonitrile (Figure 8) [108,109]. Both natural and chemical fibres are potential solid substrates for the adhesion of bacteria and nutrient media that enable growth and colony development [110,111].

Both the chemical structure and morphology of fibre-forming polymers determine the hydrophilic or hydrophobic characteristics of textile fibres. In general, chemical fibres are more hydrophobic than natural fibres. Cellulose fibres are known to be hydrophilic because they contain functional hydroxyl groups that can create attractive interactions with water molecules. Polyester fibres are hydrophobic due to the absence of hydrophilic functional groups, the inclusion of aromatic benzene rings, and chain stiffness. Hydrophobicity is also a property of polypropylene fibres due to their general chemical inertness and high crystallinity [112]. Among synthetic fibres, polyamides are the least hydrophobic due to the presence of free amine groups at the ends of the polymer chains (Figure 8). The hydrophilicity/hydrophobicity of textile fibres can be influenced by chemical and morphological modifications of their surface to introduce polar or non-polar functional groups and change the surface topography [113].

**Figure 9:**
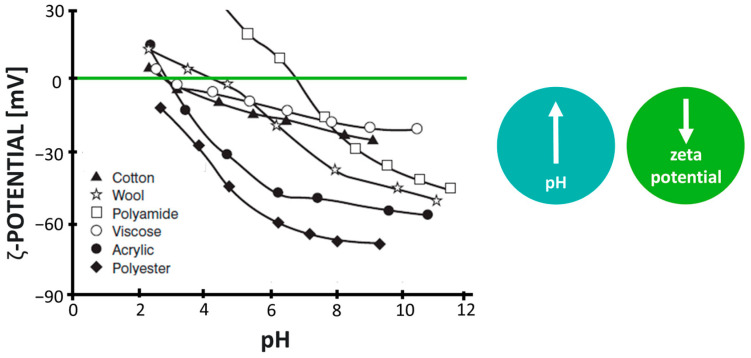
Zeta potential of different fibres depending on the pH of the medium. The green line represents the zeta potential equals zero. Reproduced with permission from [80], copyright 2006, John Wiley and Sons.

The chemical structure of the fibre-forming polymers and the presence of functional groups in their macromolecules have a major influence on the surface charge of fibres. The latter is described in terms of the electrokinetic (zeta) potential. In addition to the chemical structure, the zeta potential is also influenced by other factors of the fibre-forming polymers and the liquid medium, such as the surface polarity of the fibres, the pH value, and the ionic strength of the liquid [78,79,80,114]. When textile fibres are immersed in an aqueous solution with a pH between 6.5 and 7.0, they exhibit a negative zeta potential due to the difference in potential energy between the valence electrons of the solid and the environment (Figure 9) [80]. When the pH value increases, the zeta potential of the fibres becomes even more negative, thus ranging between −10 and −60 mV. On the other hand, the zeta potential of wool and polyamide fibres becomes positive at lower pH values, which is due to the protonation of the NH_2_ groups to the ^+^NH_3_ groups in the fibre structure. Since the negative charge is also observed in most bacterial cells in the suspension medium [111,115,116], it is generally assumed that fibres with a positive zeta potential exhibit electrostatic attraction with negatively charged bacterial cells and the fibres with a negative zeta potential exhibit electrostatic repulsion. While the former interactions are favourable for bacterial adhesion, the latter are unfavourable [111].

#### 3.1.1. Hydrophilicity/Hydrophobicity

The research results on the influence of the hydrophobicity or hydrophilicity of textile substrates on bacterial adhesion are contradictory and divide most researchers into two groups. Some authors state that the main reason for bacterial adhesion is the hydrophobicity of the polymer-based textiles [110,117,118,119], while others claim that bacterial adhesion is promoted by the hydrophilicity of the fibres [120,121,122,123].

**Figure 10:**
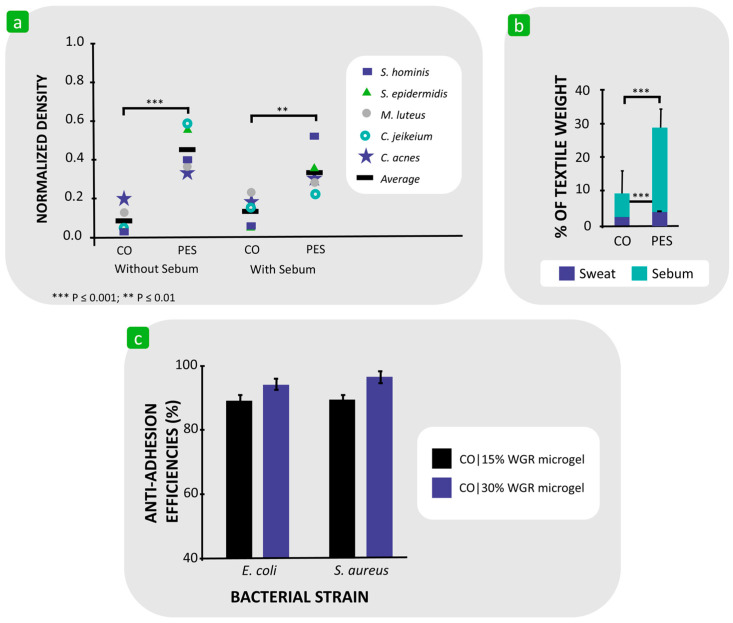
Examples showing that the hydrophobicity of textile fibres promotes bacterial adhesion. (**a**) Density of different bacterial strains adhering to cotton (CO) and polyester (PES) fibres without and with sebum. (**b**) Amount of absorbed sweat and sebum in CO and PES fibres. Reproduced with permission from [110], copyright 2021, American Society for Microbiology. (**c**) Anti-adhesion efficiency of *E. coli* and *S. aureus* on CO fabric coated with microgel of 15% and 30% WGR at 30 °C. Reproduced with permission from [119], copyright 2019, American Chemical Society.

In a study focusing on the influence of the hydrophilicity/hydrophobicity of polymer-based textiles on bacterial adhesion, Koziarz found that hydrophobic textile substrates, such as polyester, polypropylene, aliphatic polyamides, and aramids, have a greater affinity for *E. coli* K12 3300 cells than a hydrophilic cotton substrate [117]. Møllebjerg et al. also found that the bacteria *S. hominis* DSM 20328, *S. epidermidis* DSM 20044, *M. luteus* DSM 20030, and *C. jeikeium* DSM 7171 adhered better to polyester than cotton (Figure 10a) [110]. Although cotton absorbed more water than polyester, polyester absorbed significantly larger amounts of sebum, which consists of triglycerides, fatty acids, squalene, cholesterol, wax, and cholesterol esters, and dissolved sweat components (Figure 10b), and is thus a suitable nutrient source for microbial growth. It has also been suggested that the reason for the stronger bacterial adhesion to hydrophobic fibres compared to hydrophilic fibres is the lower energy barrier for adhesion. This is consistent with the extended DLVO theory, which predicts strong bacterial adhesion to hydrophobic surfaces [110,124]. Furthermore, Gu et al. found that the increased hydrophilicity of the cotton fabric, achieved by applying thermo-responsive microgels at two increased weight gain rates (WGRs), i.e., 15% and 30% WGR, resulted in anti-adhesion properties against *E. coli* and *S. aureus* (Figure 10c). It was hypothesised that the high moisture uptake of the hydrogel from the surrounding atmosphere led to the formation of a hydration layer on the fabric surface, which strongly inhibited bacterial adhesion [119].

**Figure 11:**
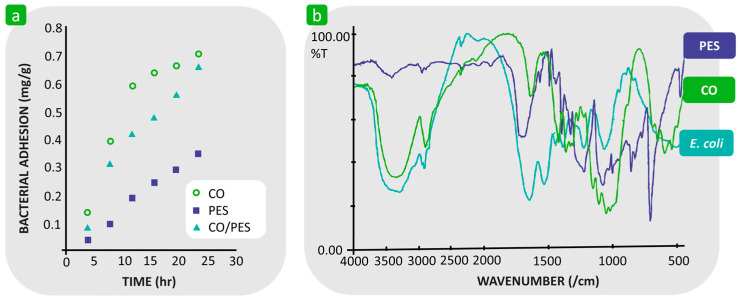
An example showing that the hydrophilicity of textile fibres promotes bacterial adhesion. (**a**) Adhesion of *E. coli* to cotton (CO), polyester (PES), and cotton/polyester blend (CO/PES) during different time periods. (**b**) FTIR spectra of PES, CO, and *E. coli* bacterial strain. Reproduced with permission from [121], copyright 2011, Elsevier.

In contrast, some authors assume that the hydrophilicity of a textile substrate promotes bacterial adhesion [120,121,122,123]. For example, Bajpai demonstrated that the adhesion of *E. coli* on hydrophilic cotton fabric is greater than on hydrophobic polyester fabric (Figure 11a) [121]. A broad and intense peak at 3344 cm^−1^ in the FTIR spectra showed that the hydrophilicity of both cotton and *E. coli* is very similar (Figure 11b), suggesting the presence of abundant hydrophilic hydroxyl groups in both cotton and *E. coli*, which could interact strongly with each other and promote bacterial adhesion. Due to the lack of hydrophilic functional groups in the structure of polyester, its attractive interactions with *E. coli* are much less effective. Their results were confirmed by the fact that bacterial adhesion on the polyester/cotton blends was significantly increased compared to 100% polyester due to the presence of cotton (Figure 11a).

**Figure 12:**
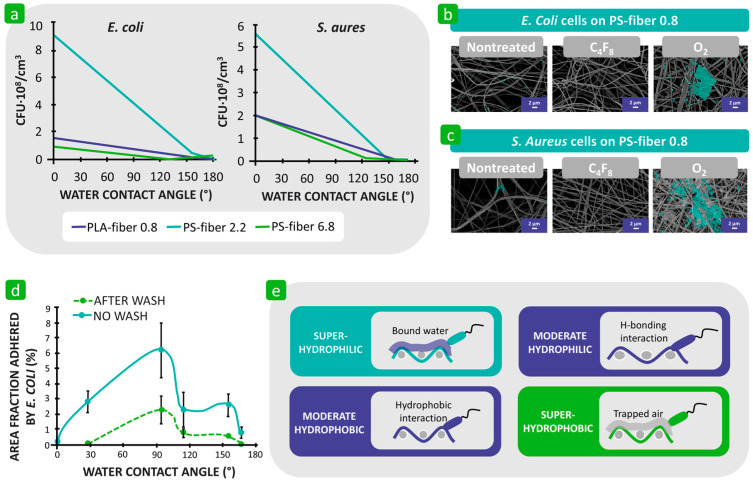
Influence of the hydrophilicity and hydrophobicity of the textile fibres on bacterial adhesion. (**a**) Adhesion of *E. coli* and *S. aureus* to different fibres as a function of water contact angle. (**b**) and (**c**) *E. coli* and *S. aureus* cells adhered to nontreated C_4_F_8_ and O_2_ plasma-treated polystyrene fibres (PS-fibre 0.8). Reproduced with permission from [123], copyright 2021, MDPI. (**d**) Average area fraction adhered by *E. coli* with respect to water contact angle on polystyrene nonwovens. Reproduced with permission from [125], copyright 2017, Royal Society of Chemistry. (**e**) Schematic representation of mechanisms of interactions.

Hemmatian et al. also found that hydrophilic fibres exhibited a higher bacterial adhesion compared to hydrophobic fibres. In this study, electrospun nonwovens of polystyrene and poly(lactic acid) were used as substrates, and their surfaces were chemically modified using O_2_ plasma to achieve a hydrophilic effect and C_4_F_8_ plasma to achieve a hydrophobic effect. The results of the bacterial adhesion correlated with the wetting properties of the samples and increased as the water contact angle on the fibre surface decreased (Figure 12a–c) [123]. In another study, Yuan et al. suggested that both extremely hydrophilic and extremely hydrophobic surfaces of polystyrene electrospun nonwovens reduce the adhesion of *E. coli* and that the nonwoven fabric with moderate hydrophobicity with a water contact angle of about 90° produces the highest level of bacterial adhesion due to the possible H-bonding and hydrophobic interactions (Figure 12d) [125]. It was emphasised that it is not hydrophobicity alone but its combination with fibre roughness and surface charge that plays an important role in the process of bacterial adhesion. The same phenomena have also been observed on other solid surfaces [125,126]. The limited bacterial adhesion on the superhydrophilic surface (water contact angle equal to 0°) was attributed to the repulsive forces between the dense water layer on the solid surface and the hydrophobic bacterial cell, as well as the possible repulsive electrostatic interactions between the same negatively charged bacteria and the solid surface [125,126]. In the case of a superhydrophobic solid surface, the attractive hydrophobic interactions were hindered because the trapped air at the interface reduced the direct contact of the bacteria with the rough fibre surface, resulting in a reduced interaction area, and thus, a lower adhesion effect (Figure 12e) [117,118].

#### 3.1.2. Combination of Hydrophilicity/Hydrophobicity and Surface Charge

**Figure 13:**
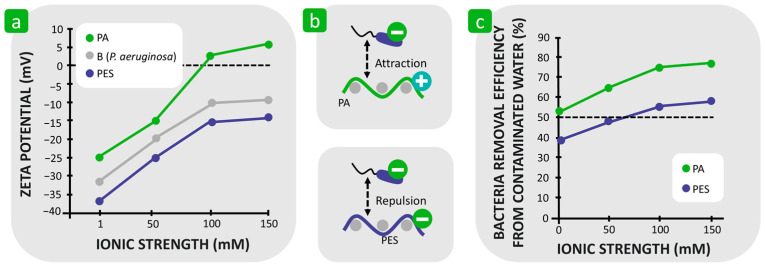
Influence of fibre zeta potential on bacterial adhesion. (**a**) Effect of ionic strength on the zeta potential of bacteria (B), polyamide (PA), and polyester (PES) fibres in the presence of calcium chloride. (**b**) Different attractions between the charged bacteria and textile substrate. (**c**) Collision efficiency as a function of ionic strength for PA and PES fibres. (**a**,**c**) were reproduced with permission from [127], copyright 2019, Taylor & Francis.

When investigating the influence of the combined factors, i.e., hydrophilicity/hydrophobicity and surface charge, on bacterial adhesion, Roy et al. found that the adhesion of the bacterium *P. aeruginosa* to less hydrophobic polyamide fibres was stronger than to more hydrophobic polyester fibres in a medium that provided a negative zeta potential for both synthetic fibres [111,127]. When the zeta potential of the polyamide fibres changed from negative to positive values at the higher ionic strength of the suspension at a pH value of 7 (Figure 13a), bacterial adhesion increased significantly. The reason for this was attributed to the attractive electrostatic interactions between the positively charged polyamide and negatively charged bacterial cells in contrast to polyester fibres, which retained a negative zeta potential under the same conditions and repelled the same charged bacteria (Figure 13b) [111,127]. Due to electrostatic attractive interactions, the efficiency of removing bacteria from contaminated water was significantly higher for polyamide than for polyester (Figure 13b). The same conclusions were reached by Zhang et al. [128], who showed that grafting inorganic basalt fibres with diethylamino functional groups to make a fibre surface more hydrophilic and electropositive led to improved initial bacterial adhesion.

The influence of the combined material factors on bacterial adhesion determined for the textile fibres was consistent with the results obtained for other solid substrates, where bacterial adhesion was highest on hydrophilic substrates with a positive surface charge, followed by hydrophobic substrates with a negative surface charge with increasing hydrophobicity, and lowest on hydrophilic substrates with a negative surface charge [81].

#### 3.1.3. Surface Free Energy

The surface free energy of textile fibres is directly related to the hydrophilicity/hydrophobicity of the fibres, as hydrophilic fibres generally have a high surface energy, and hydrophobic synthetic fibres have a low surface energy. Surface free energy, as an important surface parameter, provides valuable information about the interfacial forces and the interaction energy between the fibres and the bacteria in the adhesion process, such as the Lifshitz–van der Waals component, the electron–acceptor, and electron donor parameters, i.e., $\gamma^{LW}$, $\gamma^{+}$, and $\gamma^{-}$, respectively, which can be determined using the van Oss approach [107].

When determining the surface free energies of fibres according to Equations (8) and (9), the values obtained depend both on the method used to determine the liquid contact angle and on the liquid combinations used to calculate the surface free energy components. It should be emphasized that due to the porosity of the fibre, the contact angles of liquids smaller than 90° cannot be measured goniometrically in thermodynamic equilibrium because of the capillary rise phenomenon. In this case, the dynamic contact angles can be determined via the thin layer wicking [129,130,131]. According to this approach, the determined surface free energy of cellulose fibres is higher than 50 mJ/m^2^, whereby the electron donor component predominates [120,132,133]. For synthetic fibres, the calculated surface free energies are much lower and are in the range of 20–40 mJ/m^2^, depending on the polarity of the fibre [134,135]. The surface free energies of bacteria are between 35 and 65 mJ/m^2^ [73,136], which is a consequence of the different surface compositions of bacterial cells [137].

**Figure 14:**
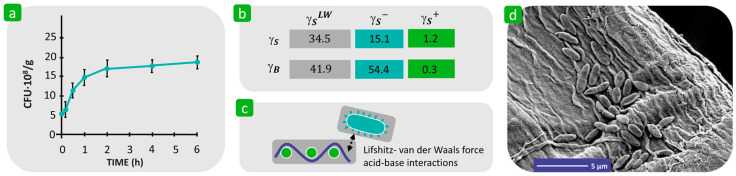
Influence of surface free energy of the textile fibres on bacterial adhesion. (**a**) The number of adherent bacterial cells on the cellulose fibres versus the incubation time in 10 mM NaCl after growth in a solution containing cellulose and NH_4_Cl in the molar ratio of 40:1. (**b**) The surface free energies of cellulose fibres ($\gamma_{S}$) and bacteria ($\gamma_{B}$) and their components. (**c**) Interfacial interactions. (**d**) SEM micrographs of *P. putida* cells adhering to the cellulose fibres (**d**). (**a**,**d**) reproduced with permission from [120], copyright 2004, Springer Nature.

There are only a few studies in the literature that report on the influence of the surface free energy of textile fibres on bacterial adhesion. Vilčnik et al. found that reducing the surface free energy of cellulose fibres from 52 mJ/m^2^ via hydrophobisation with polydimethylsiloxane to 29 mJ/m^2^ and via the combination of polydimethylsiloxane and perfluorooctyltriethoxysilane to 14.5 mJ/m^2^ significantly inhibited the adhesion of the bacterium *E. coli* ATCC 25922 to the chemically modified non-polar fabric surface, which was described as “passive antimicrobial activity” [138]. Similar results were obtained when chemically modifying cellulose fibres with fluoroalkyl-functional water-born siloxane [139]. In another study, the adhesion of *Pseudomonas putida* to paper pulp cellulose fibres was investigated. The results showed that the bacteria adhere rapidly despite the fact that both the bacteria and the cellulose fibres are highly hydrophilic and negatively charged in water, with a surface free energy of more than 50 mJ/m^2^ and with a strong electron donor character (Figure 14). The high bacterial adhesion is explained as a consequence of the action of attractive Lifshitz–van der Waals and Lewis acid–base forces, which overcome the electrostatic repulsive interactions between the same-charged bacteria and fibres. This suggestion was confirmed by the increased bacterial adhesion when the ionic strength of the liquid media was increased [120]. However, this finding agrees with the results of Hemmatian et al. but contradicts the results of Yuan et al. (Figure 12). This confirms that the bacterial adhesion process is very complex and is influenced by various factors related to the properties of the bacteria and the solid substrate as well as the environment in which the process takes place.

**Figure 15:**
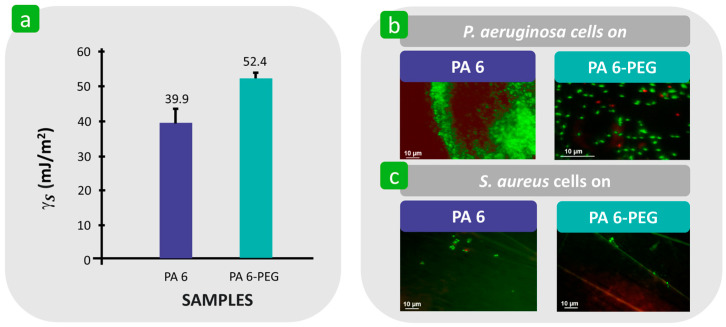
Influence of the surface free energy, $\gamma_{S}$, of the textile substrate on bacterial adhesion. (**a**) Values of $\gamma_{S}$ for polyamide 6 (PA 6) and PA 6 immobilised by PEG (PA 6-PEG). (**b**) Adhesion of *P. aeruginosa* bacterial cells to PA 6 and PA 6-PEG. (**c**) Adhesion of *S. aureus* bacterial cells on PA 6 and PA 6-PEG. The green spots indicate live bacteria; red dots represent dead bacteria. Reproduced with permission from [140], copyright 2020, MDPI.

Swar et al. reported that the immobilisation of hydrophilic poly(ethylene glycol) (PEG) to the polyamide 6 (PA 6) film increased the surface free energy from 39.9 mJ/m^2^ to 52.4 mJ/m^2^ (Figure 15a) and consequently caused a significant reduction in the adhesion of viable *P. aeruginosa* compared to unmodified PA 6, where a high amount of adherent bacteria was confirmed by the presence of green fluorescent spots (Figure 15b) [140]. The reason for this was the highly hydrated layer surrounding the ethylene oxide chain, which hindered the attraction between bacteria and the solid surface [141,142]. This observation is consistent with the results of Yuan [125]. On the other hand, the results also show that the adhesion of *S. aureus* to both surfaces was insufficient, regardless of their surface free energy. This indicates that the hydrophilicity/hydrophobicity of the solid surface is not a dominant factor influencing the adhesion properties of *S. aureus*, which has also been confirmed by other researchers [143].

**Figure 16:**
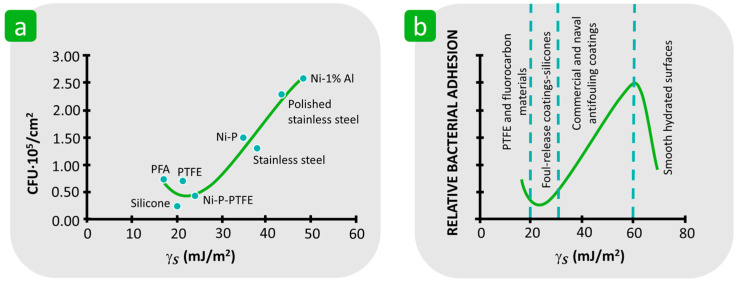
The influence of the surface free energy of inorganic solids on the bacterial adhesion. (**a**) *P. aeruginosa* AK1 retention on specific surfaces in comparison with the total surface free energy (PFA—perfluoroalkoxy alkane; PTFE—polytetrafluoroethylene). Reproduced with permission from [91], copyright 2006, Elsevier. (**b**) The Baier curve which illustrates connection between bacterial adhesion and surface free energy. Reproduced with permission from [147], copyright 2010, The Royal Society.

In contrast to textiles and other polymer substrates, the influence of the surface free energy of inorganic solids on bacterial adhesion has been studied in much greater detail. It has been shown that the $\gamma_{S}$ of solid substrates has an important influence on bacterial adhesion, which generally decreases with decreasing $\gamma_{S}$, reaching a minimum value in the range of 20 and 29 mJ/m^2^ and increasing at lower $\gamma_{S}$ [91,144,145,146,147]. For example, Pereni et al. prepared five different coatings for stainless steel with a $\gamma_{S}$ between 17.2 and 48.3 mJ/m^2^ and found that there is a non-linear correlation between the initial adhesion of *P. aeruginosa* and both the total $\gamma_{S}$ and $\gamma_{S}^{LW}$ components of the coated steel. It was shown that the maximum reduction in adhesion of *P. fluorescens* was achieved on a silicone coating with a $\gamma_{S}$ of 20.0 mJ/m^2^, followed by a nickel–phosphorus–polytetrafluoroethylene (Ni-P-PTFE) coating with a $\gamma_{S}$ of 24.2 mJ/m^2^, which was lower than the bacterial adhesion on a perfluoroalkoxyalkene (PFA) coating with the lowest $\gamma_{S}$ of 17.2 mJ/m^2^ (Figure 16a) [91]. This correlation was similar to Baier’s curve (Figure 16b) [144,148], who found that bacterial adhesion is lowest to solids with a $\gamma_{S}$ of about 25 mJ/m^2^ [148]. In another study, it was reported that the application of fluorinated diamond-like carbon coatings to stainless steel with an increased fluorine content led to a reduction in the $\gamma_{S}$ and $\gamma_{S}^{LW}$ components from 44.5 to 31.6 mJ/m^2^ and from 44.5 to 21.8 mJ/m^2^, respectively, which significantly reduced bacterial adhesion and increased bacterial removal [149].

**Figure 17:**
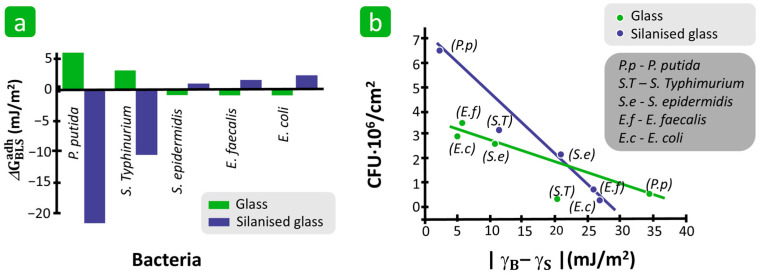
The influence of surface free energy on bacterial adhesion. (**a**) Bacterial adhesion correlated with adhesion energy, ${\Delta G}_{BLS}^{adh}$. (**b**) Surface free energy difference between the bacteria and substrate and its influence on the number of adherent bacteria. Reproduced with permission from [136], copyright 2015, American Chemical Society.

In addition, Zhang et al. suggested that not only the $\gamma_{S}$ of the solid but also the difference in surface free energy between the bacterial cell and the solid substrate is crucial for the quantitative prediction of bacterial adhesion [136]. Five bacteria, i.e., *Pseudomonas putida* KT2440, *Salmonella Typhimurium* ATCC 14028, *Staphylococcus epidermidis* ATCC 12228, *Enterococcus faecalis* ATCC 29212, and *E. coli* DH5α, and two solid substrates, i.e., glass and silanised glass, with different surface free energies were included in the experiment. While the cleaned glass surface with a $\gamma_{S}$ of 70 mJ/m^2^ was representative of a hydrophilic solid surface, the silanised glass with a $\gamma_{S}$ of 38 mJ/m^2^ was representative of a hydrophobic surface. It was found that the value of ${\Delta G}_{BLS}^{adh}$ correlates with the difference in surface free energies between the bacterial cell and the solid substrate, |$\gamma_{B}-\gamma_{S}$| (Figure 17a). The calculated adhesion energy of the hydrophobic bacteria *P. putida* with a $\gamma_{B}$ of 35.5 mJ/m^2^ was indeed high for the silanised glass with a very similar $\gamma_{S}$, but very low for the cleaned glass with a much higher $\gamma_{S}$. In contrast, the hydrophilic bacteria *E. coli* with a $\gamma_{B}$ of 65.1 mJ/m^2^ adhered strongly to the cleaned glass, but not to the silanised glass. From this, it can be concluded that the greater the difference between the surface free energies, the lower the degree of bacterial adhesion (Figure 17b). The energy difference between the substrate surface and the bacterial cells is, therefore, diminished. The graph also shows that similar differences in surface free energy do not show the same adhesion of different bacterial strains on different substrates, suggesting that hydrophilicity/hydrophobicity also plays an important role [136].

**Figure 18:**
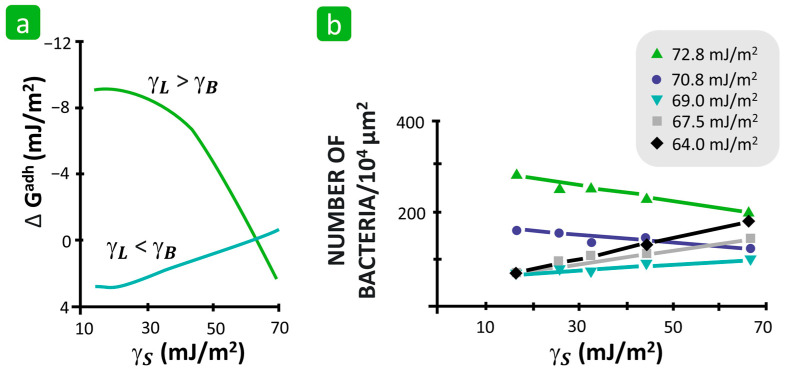
The influence of surface free energy on the bacterial adhesion. (**a**) Free energy of adhesion of a single bacterial species as a consequence of substrate free surface energy. (**b**) Bacterial adhesion as a function of substrate surface free energy for various DMSO concentrations. Reproduced with permission from [69], copyright 1983, American Society for Microbiology.

Furthermore, Absolom suggested that the ratio of the surface free energies of all three actors in the process, i.e., the bacteria, the solid substrate, and the liquid suspension medium, is crucial for the prediction of bacterial adhesion [69]. He proposed a theoretical calculation for the ${\Delta G}_{BLS}^{adh}$ of bacteria from suspension to different solid substrates as a function of their surface free energies (Figure 18a). To prove this theoretical concept, an experiment was systematically performed with five polymer surfaces with the $\gamma_{S}$ ranging from 16.4 to 66.7 mJ/m^2^, five bacteria with the $\gamma_{B}$ ranging from 66.3 to 69.7 mJ/m^2^, and five suspension media with the $\gamma_{L}$ ranging from 64.0 to 72.8 mJ/m^2^, exceeding the $\gamma_{B}$ of the bacteria, such that the liquid media exhibited both a lower and higher $\gamma_{L}$ compared to the bacteria. The results show that when the $\gamma_{L}$ of the medium was higher than the $\gamma_{B}$ of the bacteria, the value of ${\Delta G}_{BLS}^{adh}$ decreased with the increasing $\gamma_{S}$ of the solid, indicating that the bacteria preferentially adhered to hydrophobic substrates. In contrast, if the $\gamma_{L}$ of the medium was lower than the $\gamma_{B}$ of the bacteria, the value of ${\Delta G}_{BLS}^{adh}$ increased with the increasing $\gamma_{S}$ of the solid, indicating that the bacteria prefer to adhere to hydrophilic substrates (Figure 18b). This suggests that the ratio between the $\gamma_{L}$ of the liquid medium and the $\gamma_{B}$ of the bacteria determines the tendency of bacterial adhesion to hydrophobic or hydrophilic solid surfaces. Such an explanation of the mode of the bacterial adhesion process could solve the problem of contradictory statements in the literature as to whether the hydrophilicity or hydrophobicity of the solid substrate promotes bacterial adhesion. Namely, Bajpai [122], who claimed that the hydrophilicity of a textile substrate promotes the adhesion of *E. coli* to cotton, used a phosphate-buffered saline solution with a $\gamma_{L}$ of 57.2 mN/m [150] to suspend *E. coli* with a $\gamma_{B}$ of 65.1 mJ/m^2^ [136]. Since the $\gamma_{L}$ is lower than the $\gamma_{B}$ in this case, the system promotes bacterial adhesion to hydrophilic solid substrates, which is consistent with Absolom’s theoretical approach (Figure 18b). Furthermore, Koziarz’s statement that the bacterial adhesion of hydrophobic bacteria with an otherwise unknown $\gamma_{B}$ from the water medium is favoured over hydrophobic textile substrates could also be explained by Absolom’s theoretical approach, assuming that the $\gamma_{L}$ of water, which corresponds to 72.8 mN/m, is thus most likely higher than the $\gamma_{B}$ of hydrophobic bacteria [117].

### 3.2. Constructional and Textural Properties

**Figure 19:**
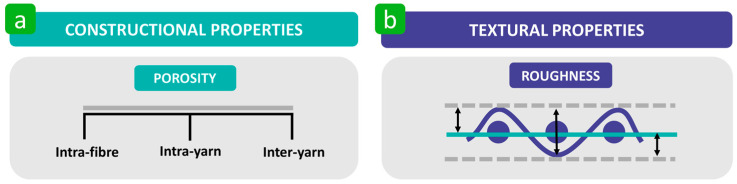
(**a**) Division of pores made accordingly to the [153]. (**b**) Schematic presentation of surface roughness.

The constructional and textural properties of polymer-based textiles are other important parameters that influence bacterial adhesion (Figure 19). Among the constructional properties, porosity is most commonly investigated. Porosity is defined as the total volume of void space within a given area of the textile substrate [151,152]. Pores can be divided into intra-fibre, intra-yarn (inter-fibre), and inter-yarn (Figure 19) [153]. In addition to constructional properties, textural properties, including roughness, are of great importance. Roughness is categorised as the highest difference in the high-frequency fluctuation range on the surface of a particular object [154,155,156]. It is quantified by assessing the deviations in the direction of the normal vector of a real surface compared to the ideal surface (Figure 19) [157,158]. There are at least 20 roughness parameters [156]. Fabric surfaces become smoother when fabric construction (threads per cm) is systematically increased. On the other hand, an increase in the linear density of yarn (diameter) increases surface roughness. In fact, there are two types of irregularities on the surface of polymer-based textiles: systematic variations (caused by uniform fabric structures, such as ribs or cords) and random variations (caused by the uneven spacing of threads or yarns) (Figure 19) [159,160]. Among the constructional and textural parameters of textile substrates that affect bacterial adhesion, roughness and porosity have been studied most intensively [76,121,122,123].

#### 3.2.1. Porosity

Different manufacturing processes for textile materials lead, among other things, to their different porosity [161]. In the case of medical implants, the porosity of the material was found to have an important influence on the level of infection, with porous materials having a higher level of infection compared to dense materials [75,162,163,164]. On the other hand, it was also found that in the case of bacterial adhesion to nonwovens, the volume and size of the pores also play an important role [123]. Bacteria prefer to live on porous surfaces whose pore size corresponds to the size of the bacterial cell, as this increases the contact between the bacteria themselves and the surface, which in turn increases the adhesion potential. The pores, which are much larger and wider compared to the size of the bacterial cell, behave similarly to a flat surface [75,165].

#### 3.2.2. Roughness

**Figure 20:**
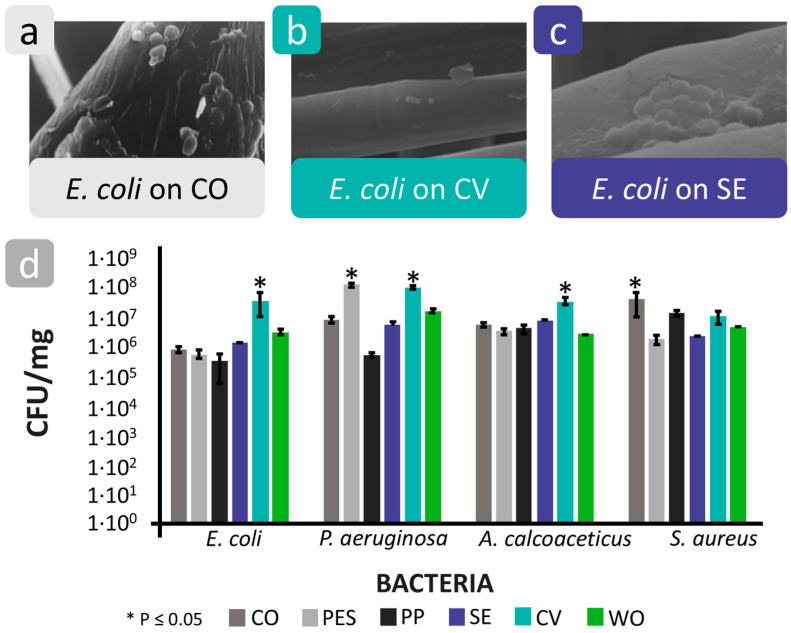
Influence of fibre roughness on bacterial adhesion. (**a**) Adhesion of *E. coli* on cotton (CO). Reproduced with permission from [121], copyright 2011, Elsevier. (**b**) Adhesion of *E. coli* on viscose (CV). (**c**) Adhesion of *E. coli* on silk (SE). (**d**) Bacterial counts on different fibres; mean count of bacterial cells in supernatant after dislodging of fibres (cotton, polyester, polypropylene, silk, viscose, wool) in PBS, incubated with different bacterial species (*E. coli*, *P. aeruginosa*, *A. calcoaceticus*, *S. aureus*). Reproduced with permission from [76], copyright 2020, Springer Nature.

The relationship between the surface roughness of polymer-based textiles and bacterial adhesion was thoroughly investigated by Bajpai et al. [121] and Varshney et al. [76] (Figure 20). Bajpai et al. [121] studied the influence of the surface roughness of cotton, polyester, and cotton–polyester blend knitted fabrics on the adhesion behaviour of *E. coli*. It was found that under the same experimental conditions, bacterial adhesion was lowest on the smooth polyester fibres, followed by the polyester–cotton blend fabric, with maximum adhesion being found on the rough cotton fibres. Furthermore, SEM analyses showed that the bacterial cells did not cover the entire surface of the fibres, but their number was higher near rough areas (Figure 20a). These results indicate that fibres with a rough surface promote bacterial adhesion. However, the results also show that the influence of textile roughness on bacterial adhesion is very complex and cannot be analysed separately from other parameters as they are directly dependent on each other. In the case of rough cotton fibres, the presence of numerous hydrophilic hydroxyl groups on both cotton and *E. coli* promoted bacterial adhesion due to strong attractive interactions. In contrast, bacterial adhesion was not supported by the hydrophobic polyester fibres with the lack of free hydroxyl groups.

Varshney et al. [76] investigated the adhesion of four bacteria, *S. aureus*, *Acinetobacter calcoaceticus*, *E. coli*, and *P. aeruginosa*, on six fibres with different nanoroughness characteristics, namely cotton, polyester, polypropylene, silk, viscose, and wool. The results show that the nanoroughness of the fibres favours bacterial adhesion. Based on the bacterial abundance on the different fibres, it was found that, in general, the maximum bacterial adhesion with a number in the order of 10^7^ to 10^8^ CFU/mg was observed on viscose fibres, with the highest nanoroughness and the lowest bacterial adhesion with a number in the order of 10^6^ to 10^7^ CFU/mg being observed on silk fibres with the lowest nanoroughness (Figure 20b–d). The SEM analysis also showed that the presence of crevices and grooves on the viscose fibres provided a larger surface area, which is advantageous for bacterial adhesion. A smooth and cylindrical surface of the silk fibres did not favour bacterial adhesion.

#### 3.2.3. Combination of Roughness, Porosity, and Hydrophilicity/Hydrophobicity

**Figure 21:**
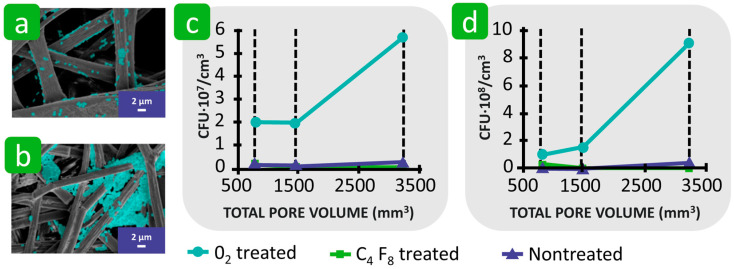
Influence of combined textile parameters on bacterial adhesion. (**a**) *S. aureus* adhesion on hydrophilic nonwoven substrate with higher mean fibre diameter. (**b**) *S. aureus* adhesion on hydrophilic nonwoven substrate with lower mean fibre diameter. (**c**) *E. coli* adhesion on the nonwoven substrates with a varied pore volume. (**d**) *S. aureus* adhesion on the nonwoven substrates with a varied pore volume. Reproduced with permission from [123], copyright 2021, MDPI.

**Figure 22:**
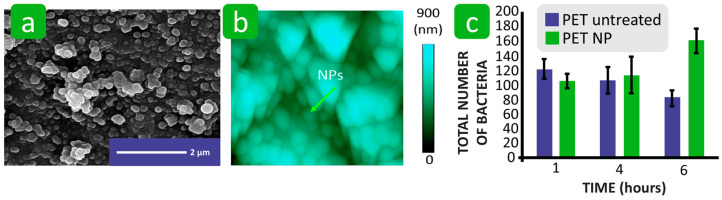
Influence of superhydrophobicity on bacterial adhesion. (**a**) SEM images of superhydrophobic polyester treated with nanoparticles (NPs). (**b**) AFM images of superhydrophobic polyester treated with nanoparticles (NPs). (**c**) Number of bacteria attached to untreated (PET UN) and superhydrophobic polyester (PET NP) samples after incubation for 1, 4, and 6 h. Reproduced with permission from [169], copyright 2023, MDPI.

It has been shown that textile parameters such as roughness, porosity, and wettability cannot be analysed separately in connection with bacterial adhesion, as they are interdependent [123,166]. In the study by Hemmatian et al., nonwovens with different fibre diameters and porosities were produced from polylactic acid and polystyrene and the bacterial adhesion of *E. coli* and *S. aureus* was investigated and compared with polylactic acid and polystyrene films. The results show that the roughness and porosity of the surface of the hydrophilic nonwovens (treated with oxygen plasma) promote bacterial adhesion compared to the films with a smooth surface and no porosity. In the nonwoven with a larger fibre diameter, most bacteria were found on the surface, and only a few were found in the spaces between the fibres (Figure 21a). Nonwovens with fibres in the submicron range showed a relatively high number of bacterial cells both on the surface and in the pores (Figure 21b). The compact spatial distribution of the fibres restricted the penetration of bacterial cells into the pores and reduced the total adhesions. The total pore volume and pore size distribution had a greater influence on bacterial adhesion than the porosity percentage. The larger the pore volume, the more bacteria adhere to the nonwoven (Figure 21c,d). Therefore, a high packing density and small pores prevent deep loading with bacterial cells and thus reduce the bacterial retention of the material. Despite the importance of surface roughness and porosity for bacterial adhesion, the wettability of the textile materials proved to be the most important factor. Thus, the superhydrophobic nonwovens (treated with C_4_F_8_ plasma) with a low total pore volume and smaller pore size would hinder bacterial adhesion [123]. It is also suggested that in superhydrophobic solid materials, surface roughness also contributes to reduced bacterial adhesion due to the combination of the chemical composition and topography, with air bubbles trapped in the nanoscale rough surface minimising the interaction area between the solid substrate and the bacteria [126,167,168]. However, some contradictory results are also reported in the literature, in which the chemical modification of polyester fibres to create a superhydrophobic and significantly rough surface did not hinder, but promoted, bacterial adhesion compared to the untreated, less hydrophobic, and smoother polyester (Figure 22) [169]. This phenomenon was explained by the increased bacterial contact within the surface microtopographies, suggesting that surface topography and roughness are crucial factors involved in bacterial adhesion.

**Figure 23:**
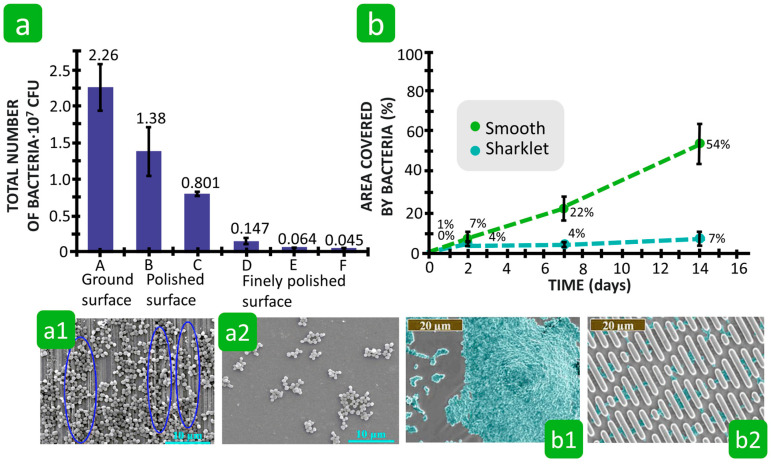
The influence of the roughness and topography of inorganic and organic solid surfaces on bacterial adhesion. (**a**) Total number of *S. aureus* adhering to the surface of the ceramic samples with different roughness. (**a1**) Representative SEM image of *S. aureus* adhesion on the ground surface. (**a2**) Representative SEM image of *S. aureus* adhesion on the fine polished surface. Reproduced with permission from [176], copyright 2020, Elsevier. (**b**) Mean value of percent area coverage of bacteria *S. aureus* on smooth and Sharklet AF™ modified surfaces of silicone elastomer at various time points (bars represent ± standard error, *n* = 5). (**b1**) Representative SEM image of *S. aureus* on smooth surface after 14 days of contact. (**b2**) Representative SEM image of *S. aureus* on Sharklet AF™ (Sharklet Technologies, Inc., Aurora, USA), surface after 14 days of contact. Reproduced with permission from [181], copyright 2007, American Vacuum Society.

Compared to textile fibres, the influence of the roughness and topography of other organic and inorganic solid surfaces on bacterial adhesion has been studied in much greater detail in order to develop materials with bacterial anti-adhesion properties [126,163,165,170,171,172,173,174,175,176,177]. Various chemical and physical fabrication technologies have been used to create topographically modified surfaces with controlled textures and pattern structures ranging from micro- to nanoscale dimensions [126]. According to the literature, the scale of surface roughness and topography, i.e., micro-, nanoscale, or hierarchical micro- to nanoscale, dictates the interaction energy between the bacterial cell and the solid surface and, consequently, the bacterial adhesion force [175]. When surface roughness and topographic features are at the microscale, with dimensions comparable to the size of bacteria, some contradictory results and interpretations are reported about the influence of surface roughness and surface topography on bacterial adhesion. The first states that rough surfaces are colonised faster than smooth surfaces because more surface area is available for adhesion (Figure 23a) [163,172,176,178,179,180]. It is assumed that the bacteria initially accumulate in the depressions of the rough surfaces, as these offer more favourable locations for colonisation due to the protection of the bacteria from environmental factors [163,178]. When surface roughness decreases, the anchoring points for bacterial adhesion decrease. Therefore, creating a smooth surface with nanoscale roughness and uniform surface textures can effectively inhibit bacterial adhesion [176]. On the other hand, the topography of a microtextured surface has also been shown to be able to reduce bacterial adhesion (Figure 23b), as the raised, textured features can potentially reduce the surface area exposed to bacteria, on the one hand, and the protruding features of the topographic surface provide a physical barrier to bacterial migration on the other [181,182,183,184,185]. Therefore, carefully designed microscale surfaces are an effective approach to limit bacterial adhesion and biofilm formation.

In addition, it has been demonstrated that the production of spatially organised microtopographic surface patterns with a roughness in the nanometre range causes a significant reduction in bacterial adhesion compared to smooth control samples due to the reduced contact area [174,175]. The results also show that topographies with nanometre-scale roughness, whose dimensions are significantly smaller than the size of the cells, effectively inhibit bacterial adhesion compared to those with microscale surface roughness. It is assumed that the spatially organised microtopographic surface patterns with nanometre-scale roughness represent a new strategy for the production of materials that resist bacterial adhesion [175]. In combination with the generated nanoroughness, a hydrophobic coating could create surface superhydrophobicity, further suppressing the adhesion of bacteria due to the trapped air in the rough hydrophobic surface and minimising the effective surface area for contact with bacteria [125,126,186,187,188,189,190]. In general, surfaces with nanoscale roughness provide better anti-adhesion properties than smooth surfaces or surfaces with larger scale roughness [190]. However, it should be emphasised that bacterial adhesion is directly influenced by other factors besides material properties, including the properties of the bacteria and the environment [183,191,192,193]. The complexity of the topic leaves a lot of room for further research.

## 4. Conclusions and Outlook

This review discusses the main chemical, physicochemical, constructional, and textural factors of polymer-based textiles in relation to bacterial adhesion. The literature data show that this relationship is very complex, as it is not only influenced by the properties of textile materials, but involves the interplay of textile, bacterial, and environmental properties. Therefore, various contradictory explanations have been presented by researchers, indicating the need for further investigation.

As far as textile factors are concerned, the influence of the hydrophilicity/hydrophobicity of textile fibres on bacterial adhesion has not yet been clarified. It is still controversial whether bacterial adhesion is promoted by the hydrophobicity or hydrophilicity of fibres. The advocacy for strong bacterial adhesion to hydrophobic fibres is supported by the lower energy barrier for adhesion, which is consistent with the extended DLVO theory. In contrast, the finding that bacterial adhesion is promoted by the hydrophilicity of fibres is explained by the strong attractive interactions between the hydrophilic hydroxyl groups of both the cellulose fibres and *E. coli* bacteria. Another study also suggests that both superhydrophilic and superhydrophobic fibre surfaces reduce bacterial adhesion and that fibres with moderate hydrophobicity generate the highest bacterial adhesion. While the limited bacterial adhesion on the superhydrophilic surface was attributed to the repulsive forces between the dense water layer on the solid surface and the hydrophobic bacterial cell, a lower adhesion effect on a superhydrophobic solid surface is attributed to the hindrance of the attractive hydrophobic interactions by the trapped air at the interface, which reduces direct contact between the bacteria and the rough fibre surface.

It has also been shown that the hydrophilicity/hydrophobicity of the textile surface is not a dominant factor influencing adhesion properties and that the combination of hydrophilicity/hydrophobicity and the surface charge of textile fibres and bacteria is crucial for bacterial adhesion. Namely, bacteria adhere much faster to less hydrophobic polyamide fibres with a positive zeta potential than to more hydrophobic polyester fibres with a negative zeta potential. The reason for this was attributed to the attractive electrostatic interactions between positively charged fibres and negatively charged bacterial cells, in contrast to fibres with a negative zeta potential, which repel the same charged bacteria. It can, therefore, be concluded that a promising strategy to prevent bacteria from adhering to textile substrates could be the development of textile surfaces with superhydrophobic properties and a negative zeta potential.

In a few studies on the influence of the surface free energy of textile fibres on bacterial adhesion, it has been reported that lowering the surface free energy of textile fibres and creating a non-polar fabric surface significantly inhibits bacterial adhesion. It was also observed that the bacteria quickly adhere to the hydrophilic cellulose fibres with a strong electron donor character, which is explained by the effect of attractive Lifshitz–van der Waals and Lewis acid–base forces that overcome the electrostatic repulsion interactions between the same-charged bacteria and fibres.

Among the constructional and textural properties of polymer-based textiles, roughness and porosity are the most important factors influencing bacterial adhesion. Hydrophilic fibres with a rough surface favour bacterial adhesion due to the presence of crevices and grooves that provide a larger surface area and increase contact between bacteria and the surface. However, the results also show that the influence of textile roughness on bacterial adhesion is very complex and cannot be analysed separately from other parameters, such as porosity and hydrophilicity/hydrophobicity, as they are directly interdependent. It is reported that the rough and porous surface of a nonwoven polystyrene promotes bacterial adhesion compared to the smooth surface of a polystyrene film without porosity. A reduced pore volume and reduced pore size prevent deep loading with bacterial cells and thus reduce bacterial adhesion. In contrast to hydrophilic textile fibres, where roughness favours bacterial adhesion, the nanoroughness of superhydrophobic fibres could contribute to reduced bacterial adhesion. This is due to the combination of chemical composition and topography, which minimises the interaction surface between the solid substrate and the bacteria.

Following the results presented in this review, future studies are urgently needed to gain insight into the basis, i.e., the influence of individual textile factors in relation to bacterial adhesion, as there have been various contradictory explanations. Whenever these findings will be clear enough, the next step is to close the missing gap in the research of combined physicochemical, constructional, and textural textile factors. In addition to the textile factors, the properties of the bacteria and the environmental characteristics in relation to bacterial adhesion to polymer-based textiles should also be investigated, which requires interdisciplinary work. Only a complex investigation of all influencing factors could finally provide valuable information for the development of new strategies and guidelines for the design of anti-adhesive textile surfaces. The idea guiding the manufacturers of such polymer-based textiles should be to shift their focus from the end of the production process, which involves the application of various finishes, as is common today, to the beginning of the textile planning process. By considering the basic principles that have been systematically outlined, simplified, and visualised in this review article, the surface interactions could be controlled to design textile surfaces to resist bacterial adhesion and biofilm formation, which is crucial for applications in medical textiles and other environments susceptible to microbial contamination.

## Figures and Tables

**Table 1:** Characteristics of the different mechanisms of bacterial adhesion.

Mechanism	Characteristics of the Phase	Characteristics of the Bacterial Cells Involved	Forces/Interactions Involved
**Reversible** **adhesion**	Immediate attraction of planktonic bacteria to a substrate surface [54,58,60,61,66].	Passive and/or active movement, but Brownian motion is still present [54,58,60,61,66].	Attractive (van der Walls) and repulsive (electrostatic) physicochemical forces [60,61].
**Irreversible** **adhesion**	Bacteria adhere firmly to the substrate surface [58,60]. The bacterial cells can also adhere to each other and form aggregates on the substrate [60].	Bacteria no longer show Brownian movement [58,60]. Production of exopolysaccharides that form a complex with the substrate surface [60].	Molecular and cellular interactions and the production of specific adhesin molecules [58,60].

**Table 2:** Overview of the three theories (DLVO, thermodynamic, and extended DLVO) of bacterial adhesion.

Theory	Strengths	Limitations	Applications
**DLVO theory**	Bacterial adhesion is assumed to be balanced by two main forces between the bacterial cells and the substrate surface [18,29,72,73,74,75].	Bacteria are more complex than inert spherical colloid particles [29,90,91]. Does not consider various factors, which also influence bacterial adhesion [29,90,91].	Only applicable when a new cell-substrate interface is formed [75]. Great importance in colloid and surface chemistry [71].
**Thermodynamic** **approach**	Explains bacterial adhesion to solid substrates based on the interfacial free energy balance [18,29,69,92].	The approach requires the estimation of the numerical values of the thermodynamic parameters [75]. Assumes a closed energy system although bacteria are living organisms [75].	Different thermodynamic approaches can be used to calculate Gibs free energy of the system [94].
**Extended DLVO** **theory**	Generally, predicts adhesion and its reversibility more accurately than the DLVO theory and the thermodynamic approach [29,75,89,105].	The theory was not rigorously tested [75].	It allows for more accurate predictions and effective solutions in managing bacterial contamination on textile surfaces.
